# Design of a Randomised Controlled Trial (RCT) on the effectiveness of a Dutch patient advocacy case management intervention among severely disabled Multiple Sclerosis patients

**DOI:** 10.1186/1472-6963-10-142

**Published:** 2010-05-27

**Authors:** Klaske Wynia, Coby Annema, Hans Nissen, Jacques De Keyser, Berrie Middel

**Affiliations:** 1 Department of Neurology, University Medical Center Groningen, University of Groningen, Groningen, the Netherlands; 2 Wenckebach Institute, School of Nursing and Health, University Medical Center Groningen, University of Groningen, Groningen, the Netherlands; 3 Graduate School for Health Research (SHARE), University Medical Center Groningen, University of Groningen, Groningen, the Netherlands; 4 Department of Home health care, University Medical Center Groningen, Groningen, The Netherlands; 5 Department of Health Sciences, University Medical Center Groningen, University of Groningen, Groningen, the Netherlands; 6 Department of Oral Health and Clinical Epidemiology, University Medical Center Groningen, University of Groningen, Groningen, the Netherlands

## Abstract

**Background:**

Case management has been suggested as an innovative strategy that facilitates the improvement of a patient's quality of life, reduction of hospital length of stay, optimization of self-care and improvement of satisfaction of patients and professionals involved. However, there is little evidence about the effectiveness of the patient advocacy case management model in clinical practice.

Therefore, the objective of our study was to examine the effects of the Dutch patient advocacy case management model for severely disabled Multiple Sclerosis (MS) patients and their caregivers compared to usual care.

**Methods/design:**

In this randomized controlled trial the effectiveness of casemanagement on quality of life of patients and their caregivers, quality of care, service use and economic aspects were evaluated. The primary outcomes of this study were quality of life of MS-patients and caregiver burden of caregivers.

Furthermore, we examined quality of life of caregivers, quality of care, service use and costs.

**Discussion:**

This is a unique trial in which we examined the effectiveness of case management from a broad perspective. We meticulously prepared this study and applied important features and created important conditions for both intervention and research protocol to increase the likelihood of finding evidence for the effectiveness of patient advocacy case management. Concerning the intervention we anticipated to five important conditions: 1) the contrast between the case management intervention compared to the usual care seems to be large enough to detect intervention effects; 2) we included patients with complex care situations and/or were at risk for critical situations; 3) the case managers were familiar with disease specific health-problems and a broad spectrum of solutions; 4) case managers were competent and authorized to perform a medical neurological examination and worked closely with neurologists specialized in MS; and 5) the case managers had a regional network of professionals and health care organisations at their disposal, and were accepted as a coordinator of care. We also put a lot of effort on the selection of eligible patients, randomization and statistical methods, but also on power analysis, selection of reliable, validated and sensitive outcome measures, and (statistical) control of confounders.

**Trial registration:**

Dutch Trial Register http://www.trialregister.nl. Trial ID: NTR762.

## Background

### Case management

Case management has been suggested as an innovative strategy that facilitates the improvement of a patients' quality of life, the reduction of hospital length of stay, the optimization of self-care and improvement in the satisfaction of the patient and the professionals involved.[1,2] In general, case management focuses on high-risk and high-cost populations that represent the largest part of the expenses for health care in developed countries.[3,4]

In case management, an individual or a small team is responsible for navigating the patient through a complex process in the most efficient, effective and acceptable way.[5] Based on suggestions of the underlying dynamics, Long [6,7] categorized the many variants of case management into two types: the "interrogative case management model" and the "patient advocacy" model:

▪ The predominant focus of the interrogative case management model is on the appropriateness of services during the initial clinical decision-making process. The costs of care in particular are recognized as representing a legitimate argument in this process. This model, also referred to as the "medical case management model" [8] and the "gate-keeper model" [9] employs a physician gatekeeper with expectations of cost containment by arranging substitution of services.

▪ The predominant focus of the patient advocacy case management model is on a more comprehensive coordination of services across the continuum of care, viewed from the patient perspective.[10] The case manager assesses the changing needs of the clients, eliminates fragmented care, and arranges for services to be provided.[7] In this model, also referred to as the socioeconomic model, the treatment regimen is determined not only by the medical needs, but also by a combination of financial, psychological and social circumstances of the patient.

The case management approach in the patient advocacy model seems especially relevant for the growing number of chronically ill and elderly people with complex health problems, and is in line with the contemporary general emphasis on patient-centered health care and the delivery of effective health care for these patients.[11]

### The Dutch case management model for the chronically ill

In the Netherlands we developed a case management model for people with a chronic disease in a multidisciplinary and integrated care setting.[12] This model was developed using literature review, four focus group meetings (n = 6), and a Delphi method (n = 65) with two written rounds and a consensus meeting. The participants of the Delphi panel were patients and caregivers (27%), medical and non-medical professionals (51%) and health administrators (23%).

The final model consists of five basic assumptions and 21 characteristics and was evaluated by 92% (n = 58) of the Delphi panel as (very) desirable for the chronically ill. The characteristics of the Dutch case management model for the chronically ill are similar to the characteristics of the patient advocacy model. Most important characteristics of the Dutch model are: (i) the patient-centred vision, (ii) the focus on somatic as well as the psychosocial and environmental problems, and (iii) the independency of the case manager from organizational and professional structures. In fact the patient - case manager relation can be seen as an employer - employee relationship in which the case manager acts under agreement of the patient.

### Effectiveness of case management

There are several reasons to perform studies to evaluate the efficacy of the patient advocacy model. The first is that financial constraints require that we select effective interventions that at the least maintain quality of care. A second reason is the growing number of chronically ill and elderly people with complex health problems. These groups in particular seem to benefit from the patient advocacy model in which the case manager assesses the changing needs of the clients, eliminates fragmented care, and arranges for services to be provided[7]. Although it is expected that less costly appropriate substitute services are likely to be used whenever possible[7], it is important to know which effects the patient advocacy model has on service use and health care costs.

There is little evidence about the effectiveness of patient advocacy case management model in clinical practice. However, Oeseburg et al[13] showed in a systematic review of randomized controlled trials (RCTs) that the patient advocacy case management model did not increase service use and costs. Moreover, there were indications that patient advocacy case management for chronically ill or elderly people may lead to a decrease in service use and health care costs. Another conclusion was that little attention was paid to other potential benefits of patient advocacy case management such as physical health or quality of life aspects. These potential limitations tend to result in a narrowed view of the effectiveness of patient advocacy case management.

### Multiple Sclerosis (MS)

In this study we examine the effects of patient advocacy case management among severely disabled MS patients. MS is an inflammatory autoimmune disease of the central nervous system and is potentially the most common cause of neurological disability in young adults.[14] After diagnosis the prognosis of the disease is difficult to estimate and is dependent on its course. In fact, on the individual level, MS is an unpredictable chronic disease with a severe impact on functioning and quality of life. MS-patients can benefit from the patient advocacy model when the disabilities and care complexity increase. Consequence of the increasing care dependency is a growing burden and strain for the caregivers, the persons directly involved in the care for the MS-patients. When care-dependency increases the decision between living at home and living in a nursing home is often based on the caregiver burden and strength.

### Objective

The objective of our study was to examine the effects of the Dutch patient advocacy case management model for people with a chronic disease compared to usual care on (i) quality of life of patients and their caregivers, (ii) quality of care, (iii) service use and economic aspects.

Expected outcomes of patient advocacy case management compared to the usual care were: (i) improved quality of life for both, patients and caregivers (primary outcome patients), (ii) improved quality of care, (iii) less caregiver burden or strain (primary outcome caregivers), and (iv) equal or less service use and costs.

This paper will provide a detailed description of the study design and research protocol.

## Methods/design

### Study design and setting

The study was a randomized controlled trial performed among severely disabled MS-patients known to the Groningen MS center, and their caregivers. Study participants received either case management according to the Dutch patient advocacy case management model or the usual care during the intervention period of 15 months between September 2006 and January 2008.

### Randomisation procedure

Eligible patients (and their caregivers) were randomly allocated - with a computerized programme - to either the intervention group or the control group using a randomized block design.[15] This approach allowed us to control our findings for characteristics of the participants. Blocking variables in our study were severity of limitations in walking (yes/no wheelchair dependency), having a partner or caregiver (yes/no), educational level (low/middle/high level), having children living at home (yes/no), and performing paid work (yes/no).

### Ethical approval

The study has been approved by the University Medical Center Groningen medical ethical committee (Reference M06.040514).

### Study population MS-patients

The study was performed among MS-patients known to the Groningen MS center, which is part of the University hospital. Patients lived in a wide surrounding area around the hospital with a maximum distance of about 100 kilometres. As it is known that case management is most effective in high-risk populations, eligible patients should be characterized by complex care situations and/or be at risk for exacerbations of critical situations. We therefore included:

▪ MS-patients with increasing limitations in walking ability who were at risk for becoming completely dependent of a wheelchair and help from others. This phase is especially complex because of the (potential) changes in psychosocial circumstances and necessary environmental changes. Furthermore, this phase is critical for partnerships because of the changing roles between the partners and the growing caregiver burden.

▪ MS-patients that were completely wheelchair dependent but not essentially restricted to bed much of day. This phase is especially complex because of the growing risk of physical complications and care dependency. This phase is also critical for the caregiver because of increasing caregiver burden. In summary, admission to a nursing home is a threatening solution for all health-related and caregiver problems.

To define our sample of severely disabled MS-patients we used the neurological classification system, the Expanded Disability Status Scale (EDSS). The EDSS is a commonly used disability measure for MS[16] with a twenty-points scoring scale starting at 0 (no disability) and increasing at half point increments to a maximum score of 10 (death due to MS).

Eligible patients with increasing limitations in walking were defined as patients with an EDSS score ranging from 4.5 to 7.0 (= able to walk without rest some 300 meters or less without assistance or with canes or other assist devices). Patients essentially restricted to wheelchair but not essentially restricted to bed much of day were defined with an EDSS score ranging from 7.0 to 8.5). Furthermore, patients should be of adult age, live independently in the community and not in a nursing home or other institution for long term referral. Patients should not participate in another intervention study (Table 1).

**Table 1:** Eligibility criteria for the Dutch patient advocacy case management study.

Inclusion criteria
- Adult people older than 18 years - Diagnosed with MS - EDSS score with a range of 4.5 to 8.5 - Living at home - Treated by neurologists of the Groningen MS centre, which is part of the University hospital

**Exclusion criteria**

- No clear diagnose for MS - EDSS score below 4.5 or above 8 - Living in a nursing home or other institution for long term referral - Participation in another MS related study - Living at a larger distance from the hospital than 100 kilometers

In total 227 eligible patients with the diagnosis MS and a suitable EDSS score were selected from our hospital registration system and invited by mail to participate in the study according to the Informed Consent procedure in our study protocol. Patients were informed about the intervention, the intervention period, the fact that there was an equal chance to get allocated to the case management group or to the usual care group, and the efforts asked by the researchers to fill out a number of questionnaires at the start and at the end of the intervention period of 15 months.

**Figure 1:**
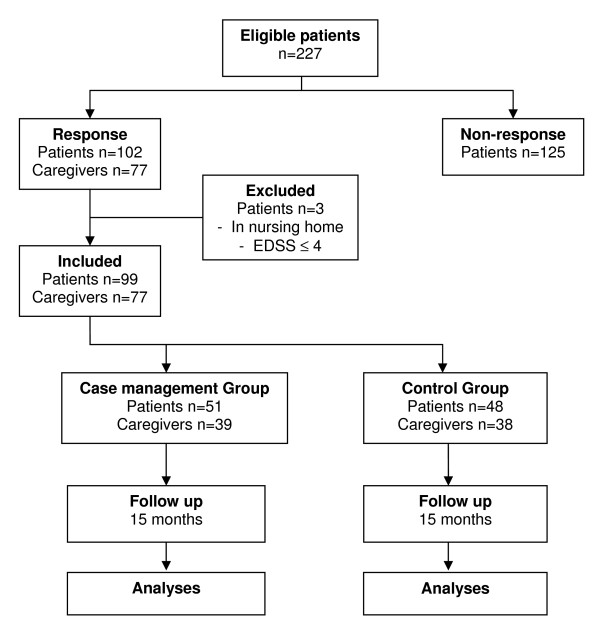
**Flowchart Dutch patient advocacy case management intervention among severely disabled MS-patients**. Summary of the progress through the phases of the study until the allocation of the participants to each treatment arm.

Finally, 102 MS-patients (45%) responded to our invitation to participate in our study. There were no differences between respondents and non-respondents concerning age (T-test p-value .332) and gender (Mann-Whitney Test p-value .374). Respondents answered the questions necessary to check the inclusion criteria and for the randomized block procedure. From these questions - and from telephone calls by the researcher - it became clear that three patients were living in a nursing home or had no walking disabilities. In total, 99 patients (44% response) agreed to participate in the study and signed the Informed Consent paper including the consent for the case manager to perform the usual neurological evaluation under supervision of the neurologist, to read the medical dossier, and to contact the general practitioner when necessary. The computerized randomization programme assigned 51 patients to the case management group, while 48 patients were included in the usual care group (Figure 1).

### Study population Caregivers

Caregivers of participating MS patients were invited to participate in the study according to the Informed Consent procedure in our study protocol. Caregivers were informed about the intervention, the intervention period and the special attention of the case manager for the caregiver burden, the fact that there was an equal change to get allocated to the case management group or to the usual care group, and the efforts asked by the researchers to fill out a number of questionnaires at the start and at the end of the intervention period. In total, 77 caregivers agreed to participate in the study and signed the Informed Consent paper: Thirty-nine caregivers were included - with the MS-patients they were related to - in the case management group and 38 caregivers were included in the usual care group (Figure 1).

### Case managers

Case management was performed by two experienced professionals in care-coordination for people with MS. One case manager was a nurse practitioner of the MS center of our university hospital while the other case manager was a nurse specialist in chronic neurological diseases especially in MS of a large home healthcare organisation. Both case managers were familiar with disease specific health-problems and a broad spectrum of solutions for these problems. Furthermore, both case managers were competent and authorized to perform a medical neurological examination and worked closely with the neurologists specialized in MS.

Both case managers had a regional network of professionals and health care organisations at their disposal. Because of their years of experience in the regional MS-care in the community and hospital the acceptance of the case manager as the coordinator for care delivery was accepted. The case managers were not limited to organizational boundaries in allocating services. Patients' preferences were leading in the selection of the most appropriate service.

### Intervention: Dutch Patient Advocacy Case management

Elements of the Dutch Patient Advocacy Case management model with regular home visits were:

▪ Use of valid and reliable self-report assessment tools: The Multiple Sclerosis Impact Profile (MSIP) [17,18] for the disease specific health-status of the patients, and the Caregiver Strain Index (CSI) [19] to assess the caregiver burden. In the paragraph about outcome measures these assessment tools are described.

▪ Regular assessments (each five months, three times during the study) during a visit at the patients' home: Patients prepared themselves by filling-out the MSIP. Caregivers prepared themselves by filling-out questions concerning caregiver burden. Casemanagers prepared themselves by reading the filled-out questionnaires. During the assessment the filled-out questionnaires were guidelines for the discussion for the patient, caregiver and case manager.

▪ Medical examination and treatment: During each visit the case managers also performed a medical examination as a part of the total assessment. Concerning neurological medical aspects, like changes in medical treatment, the case managers were supervised by the neurologists of the MS centre structurally (during weekly meetings) and incidentally (when necessary). For regular medical treatment the case managers contacted the patients' general practitioner.

▪ Care plan: using the outcomes of the assessments, priorities set by the patient and caregiver and advise for possible solutions given by the case manager, a multidisciplinary and patient-centred care plan was composed. Health problems, preferred solution directions, actions to be undertaken and agreements about who is doing what and when were registered in the care plan.

▪ Navigation and monitoring: after the care plan was established and put into action, there were regular contacts between the case manager and the patient and caregiver about the progress in the realization of the plan and the effects on patient and caregiver. These contacts were mostly telephone contacts and sometimes additional home visits.

▪ Evaluation: during each follow-up home visit the care plan was evaluated and results were described.

### Usual Care: regular consultations in the hospital

Patients and caregivers in the control group received the usual care during regular consultations with their neurologist in the Groningen MS center. Normally, patients visited their neurologist about two times a year. This frequency may decrease or increase depending of the patients' personal preference and actual health-status. The time planned for a standard consultation was 20 minutes. Consultations focused on the neurological health problems of the patient. For non-neurological issues patients were referred to their general practitioner or other health professionals. For non-medical issues patients were referred to a nurse specialist.

### Procedure of data collection

After inclusion in the study and assignment to either the intervention or the control group, patients were informed by mail about their inclusion and allocation. Patients included in the control group were advised to make an appointment with their neurologist as they were used to do. Patients included in the experimental group were randomly assigned to one of the two case managers.

Data were collected with self-report outcome measures on quality of life (patients and caregivers), quality of care (patients) and caregiver burden (caregivers) at two moments during the study: i) at baseline, after inclusion in the study and randomization to one of the research groups, ii) 15 months after the baseline assessment a few weeks after finishing the intervention. See also Table 2.

**Table 2:** Measures

Variable	Measure	Applied to	T0	T1	Cont
Demographics	Usual questions	Patient and Caregiver	X		
Medical history	Usual questions	Patient	X		
Quality of Life	WHOQOL-BREF	Patient and Caregiver	X	X	
	MSIP	Patient	X	X	
	IPAQ BDI-II	Patient and Caregiver	X	X	
	BDI-II	Patient and Caregiver	X	X	
Quality of Care	CSQ	Patient and Caregiver	X	X	
	Care plan/Medical letter	-			X
	EQ-5D	Patient and Caregiver	X	X	
Caregiver burden	General Inventory	Caregiver	X	-	
	CSI	Caregiver	X	X	
Costs	Care plan/Medical letter	-			X
	Costs booklet	Patient			X

T0 = at baseline after before randomization. T1 = 15 months after finishing the intervention. Cont. = continuous. WHOQOL-BREF = World Health Organization Quality of Life, abbreviated version; MSIP = Multiple Sclerosis Impact Profile; IPAQ = Impact on Participation and Autonomy Questionnaire; BDI-II = Beck Depression Inventory-II; CSQ = Consumer Satisfaction Questionnaire; EQ-5D = Health related Quality of Life Index; CSI = Caregiver Strain Index

During the intervention data were collected continuously about: i) health problems, interventions and outcomes from the care plans (case management group) and the medical records (control group), and ii) about service use and costs from a structured costs booklet for the patients in both research groups and structured case management records. See also Table 2.

### Outcome measures

#### General Quality of Life (primary outcome patients)

The World Health Organization Quality of Life, abbreviated version (WHOQOL-BREF)[20] is a generic quality of life measure with a broad scope, including environmental aspects. It consists of 26 items in four constructs: 'physical health and autonomy', 'psychological health', 'social relationships' and 'environment', and two separate questions: 'overall quality of life' and 'overall satisfaction with health'. For each scale, item scores were coded, summed and transferred to a scale of 0 (worst health) to 20 (best health). In our former study among MS-patients the WHOQOL-BREF showed satisfactory levels of internal consistency with Cronbach's alpha = .63/.81[17].

#### Participation and autonomy

The Impact on Participation and Autonomy Questionnaire (IPAQ) [21,22] is a domain specific quality of life measure covering participation aspects. The IPAQ consists of twenty-five items focusing on person-perceived participation and autonomy. The instrument assesses two aspects of participation: perceived participation and the perceived problem. In this study the perceived participation aspect was used. The sub-domains are autonomy indoors, family role, autonomy outdoors and social relations. Item scores are graded on a five-point rating scale with discrete responses, ranging from 1 (very good) to 5 (very poor). Scores are summed for each domain. In a former study among MS-patients the IPAQ showed satisfactory levels of internal consistency with Cronbach's alpha = .86/.94.[17]

#### Depression

The Beck Depression Inventory-II (BDI-II) measures the severity of self-reported depression in adolescents and adults.[23,24] The BDI-II consists of 21 items reflecting the diagnostic criteria for major depressive disorders that are described in the American Psychiatric Associations Diagnostic and Statistical Manual of Mental Disorders, 4^th ^Edition (DSM-IV).[25] Respondents are asked to describe themselves for the "past two weeks, including today" by selecting a response option for each item. Item scores are graded on a 4-point scale ranging from 0 (no negative feelings) to 3 (severe negative feelings). Scores are summed and total scores can range from 0 to 64. Suggested cut-off values are the following: 0-10 = no depression; 11-17 = mild depression; 18-23 = moderate depression; 24-39 = severe depression.

#### MS-specific health-status

The Multiple Sclerosis Impact Profile (MSIP)[17] is a self-report measure for people with MS to assess disability and disability perception and is based on the International Classification of Functioning, Disability and Health (ICF). The MSIP consists of two related parts: 1) the MSIP-Disability (MSIP-D) part, reporting the prevalence and severity of MS-related disabilities and 2) the MSIP-Disability Perception (MSIP-DP) part, reflecting the perception of reported disabilities. A disability perception item is directly linked to each disability item. The MSIP consists of 36 items for each part, divided into three functioning scales: 'Muscle and movement functions', 'Excretion and reproductive functions' and 'Mental functions'; two activities scales: 'Basic movement activities' and 'Activities of Daily Living'; one participation scale: 'Participation in Life situations'; and one environmental factors scale: 'Environmental factors'; and four additional impairment items for Fatigue, Pain, Seeing functions and Speech functions. Scoring options for the MSIP-disability part range from 0 (no disability) to 3 or 4 (complete disability). Summed scores for the disability domains indicate the extent of disability. A lower disability score means less serious disability. Scoring options for the MSIP-disability perception part range from 0 (no, never) to 3 (yes, always). Summed scores for the disability perception domains indicate the extent to which patients perceive the reported disabilities as problematic. A lower disability perception score means that a reported disability is perceived as less problematic.

The internal consistency tests of MSIP scales showed good levels of internal consistency with Cronbach's Alpha's = .80/.90 for most scales, and satisfying and weak Cronbach's Alpha's for the mental functioning scales (.62/.65) and the 'Environmental' scales (.49/.50).[17,18]

#### Overall Quality of Care

The 'Client Satisfaction Questionnaire (CSQ-8)'[26] consists of eight items belonging to one domain: satisfaction with the received care. Items can be scored on a scale ranging from 1 (very negative) to 4 (very positive). The total score therefore can range from 8 (most dissatisfied) to 32 (most satisfied). The Dutch version of the CSQ-8 showed similar high internal consistency (Cronbach's Alpha = 0.91) as the original English version (Cronbach's Alpha = 0.93).[27]

#### Care plans and medical letter

Two researchers independently analyzed each care plan (case management group) and medical letter (control group) for the reported health problems of the patients, caregiver burden, applied interventions, and patient and caregiver outcomes. Differences between the researchers were resolved through discussion or with reference to a third researcher when necessary.

Findings were recorded and categorized in SPSS.

▪ Disease related health problems were categorized into the MSIP-categories;

▪ Caregiver burden problems were summarized as caregiver problems;

▪ Interventions were clustered into categories of 'giving information', 'consultation of medical and non medical specialists or organizations', 'refers medical and non medical specialists or organizations', 'acquiring assist devices or home adjustments', 'arranging supplementary care ad home' or 'arranging (short) stay or day treatment'

▪ Outcomes were recorded in terms of 'improved', 'worsened' or 'no change'

#### Opinion about case management

To examine the patients and caregivers opinion about the case management process and outcomes we developed thirty statements about case management. These statements were based on the assumptions and characteristics defined in the Delphi study in which we developed the Dutch casemanagement model [12]. Patients were asked to give their opinion about their personal experience (what case management did bring them), and the case management processes (e.g. the home visits, the care plans, the casemanagers actions). We linked four response options to each statement: I fully disagree, I disagree, I agree, I fully agree.

#### Caregiver context and tasks

To gain insight into the caregivers tasks and time spent, relevant contextual factors (age, gender, relation to MS-patient, health status), personal experiences with caregiver tasks (e.g. (not) heavy, (not) problematic) we developed an inventory. Relevant items for this inventory were selected from existing questionnaires applied in other studies about caregiver tasks and caregiver burden.

#### Caregiver Burden (primary outcome caregivers)

The Caregiver Strain Index (CSI)[28] measures caregiver burden related to care provision. The CSI can be used as a tool to identify caregivers with potential care giving burden and strain. The CSI consists of 13 items with at least one item for each of the following major domains: Employment, Financial, Physical, Social, and Time. Positive responses to seven or more items on the index indicate a greater level of strain. Positive responses on four to six items of the index indicates an increased burden, while positive responses on seven to nine items indicates a risk for overburdening or strain, and a positive response on ten to twelve index items indicates a clear overload or strain.

### Economic evaluation

For economic evaluation from the societal perspective data on service use and indirect costs were collected from several sources:

▪ Patients in both research groups filled out a structured costs booklet each three months.

▪ Casemanagers recorded travelling costs and time spent on each patient, while for comparison with the costs for usual care the standard costs for a consultation with a neurologist were used.

▪ We analyzed care plans (case management group) and the medical records (control group) for data on service use.

▪ Patients and caregivers filled out the EQ-5D for calculating the costs of an additional day of life. The EQ-5D is a health-related quality of life measure consisting of i) five dimensions (mobility, self-care, usual activities, pain/discomfort, anxiety/depression) each of which can take one of three responses reflecting three levels of severity (no problems/some or moderate problems/extreme problems) within a particular EQ-5D dimension, and ii) a standard vertical 20 cm visual analogue scale (similar to a thermometer) for recording an individual's rating for their current health-related quality of life.

### Sample size calculations

We used samples of MS-patients as well as samples of caregivers and expected that about 60% of the caregivers would give their informed consent to participate in the study. Sample size calculations were targeted on a clinically relevant change in quality of life for the smallest group, the caregivers. A clinically relevant change in the primary outcome measure of quality of life (WHOQOL-BREF) of 1.5 points on a scale ranging from 0 (worst quality of life) to 20 (best quality of life) and standard deviation of 2.0 can be found with a power of 0.815 in comparing two groups of 30 subjects is applied. The clinically relevant change of 1.5 points and standard deviation of 2.0 were found in a former study among 530 MS patients [17,29] Consequently the sample size for the MS-patients should consist of 50 subjects for each research group. Power analysis for this sample size showed a power of 0.960.

### Potential confounders and effect modifiers

To prevent confounding in the control group during the consultations in the hospital were not performed by the casemanagers involved in the project during the intervention period. For special non-medical interventions casemanagers could be consulted by the neurologist, while contacts of the casemanagers with patients from the control group were limited to that specific intervention they were asked for by the neurologist.

### Statistical analysis

Primary analysis will focus on comparison of samples and differences between the intervention group and control group after intervention concerning the primary outcome measures: quality of life for the MS-patients and caregiver burden for the caregivers. Secondary analysis will focus on: 1) the comparison between research groups for the secondary variables concerning quality of life, quality of care, service use and costs, and 2) the explanation on the outcomes on the primary outcome variables. Finally, analysis will focus on the patient-caregiver couples: e.g. the impact of the patients' disability and disability perception (MSIP scales) on the caregiver strain en caregiver quality of life.

For comparison of samples and estimation of differences in outcomes between the case management and usual care groups before and after the intervention period we used t-tests for continuous variables, Chi-square tests and Fisher exact tests when appropriate, and differences of proportions tests[30] for comparisons with categorical variables. For multiple group comparisons we will use one-way ANOVA analysis to determine statistically significant differences between subgroups. Effect sizes will be calculated only for statistically significant group differences (p < 0.05) with post hoc tests (with Bonferroni correction for capitalization on chance in multiple testing). According to Cohen's thresholds [31] an ES of < 0.20 indicates a trivial effect, an ES of ≥ 0.20 to < 0.50 a small effect, an ES of ≥ 0.50 to < 0.80 a moderate effect and an ES ≥ 0.80 a large effect. An ES ≥ 0.20 reflects a clinical relevant difference between groups.[32]

The impact of case management on quality of life (patients and caregivers) and on caregiver burden (caregivers) will be assessed using hierarchical regression analysis with each of the Quality of Life scale variables, participation scale variables and depression variable (patients and caregivers) and Caregiver Strain Index outcome variable (caregiver) as dependent variables. Relevant other patient, disease related and caregiver variables with a statistically significant correlation with the dependent variables will be include in the analysis as covariates.

The primary economic evaluation will be a cost-effectiveness analysis from the societal perspective. In a secondary cost-utility analysis, quality adjusted life years (QUALYs) will be calculated based on EQ-5D values.

## Discussion

In this paper we have reported on the background and the study protocol of a unique randomized controlled trial to examine the effects of the Dutch patient advocacy case management model. We meticulously prepared this study and applied important features and created important conditions for both intervention and research protocol to increase the likelihood of finding evidence for the effectiveness of patient advocacy case management.

Concerning the intervention we anticipated to four important conditions: Firstly, the contrast between the case management intervention - in combination with home-visits - compared to the usual care seems to be large enough to detect intervention effects. Secondly, as it is known that case management is most effective in high-risk populations, we decided that eligible patients should be characterized by complex care situations and/or should be at risk for exacerbations or critical situations. Thirdly, the case managers in this study were familiar with disease specific health-problems and a broad spectrum of solutions for these problems. Furthermore, both case managers were competent and authorized to perform a medical neurological examination and worked closely with the neurologists specialized in MS. Finally, the case managers had a regional network of professionals and health care organisations at their disposal, and were accepted in the community and in the hospital as a coordinator of care.

Apart from the intervention, the study design is of essential importance for finding evidence for the effectiveness of case management. We therefore developed a powerful design: We put a lot of effort on the selection of eligible patients, randomization and statistical methods, but also on power analysis, the selection of reliable, validated and sensitive outcome measures, and (statistical) control of confounders, and finally the assessment by a medical ethics committee.

We decided against performing a feasibility study. Arguments were that the intervention (casemanagement) was already tested on a small scale in days-to-day practice, and evaluated by patients, casemanagers and other relevant health professionals. Furthermore most applied measures, utilized in the trial, were tested in earlier MS-related studies. In other words, we acquired sufficient evidence for feasibility and based on these experiences we were confident about the adequacy of our study design and data collection plan.

A possible limitation of the study is the duration of the intervention period. There are indications suggesting that there is a so-called 'investment effect' concerning health care costs for 'steady-state programmes' such as patient advocacy case management.[33] This phenomenon was found in a number of experimental studies[2,34-36] and implicates that in the first year of a study, the health care costs in the experimental group were more than those incurred for patients in the usual care group, while in the final years of these studies costs for the usual care group exceeded those for the experimental group. Although there is no evidence it seems reasonable to assume that this investment effect for costs also is applicable for other outcome variables like quality of life. Despite our efforts made to organize a prolonged study, we succeeded in organizing an intervention period with duration of 15 months.

## Competing interests

The authors declare that they have no competing interests.

## Authors' contributions

KW was principal investigator, conceived and drafted the study, and this manuscript. CA was investigator, was involved in the design of the study, was responsible for data collection and helped to draft the manuscript. HN and JDeK commissioned the study, provided the necessary conditions, and participated in the design of the study and. BM was methodological and statistical investigator and participated in the design of the study. All authors contributed to the manuscript and have read and approved the final version of the manuscript.

## Pre-publication history

The pre-publication history for this paper can be accessed here:

http://www.biomedcentral.com/1472-6963/10/142/prepub
